# Regional differences of COVID-19 pandemic prevention in China: Especially from the perspective of political leaders

**DOI:** 10.3389/fpubh.2022.1037242

**Published:** 2022-11-04

**Authors:** Jingli Li, Jianjun Zhang, Zhiyong Han

**Affiliations:** ^1^School of Management, Henan University of Technology, Zhengzhou, China; ^2^Guanghua School of Management, Peking University, Beijing, China; ^3^School of Business Administration, Anhui University of Finance and Economics, Bengbu, China

**Keywords:** pandemic prevention, social and economic factors, coping capacity, prefectural-level leader teams, COVID-19

## Abstract

Since emergency management capabilities with respect to COVID-19 differ across different regions of China, it is necessary to adopt a more comprehensive perspective to study the reasons for these differences and propose corresponding policies. By investigating 287 prefectural-level administrative regions, this study explores the reasons for different levels of COVID-19 prevention performance. The results lead to the following conclusions. The factors influencing pandemic prevention include both structural factors, such as economic and social factors, and the experiences and characteristics of prefectural-level government leaders (party secretaries and mayors), such as grassroots work experience and level of education, which are significantly positively correlated with the progress of pandemic prevention. Based on these findings, we propose suggestions to improve governance capacity in terms of three aspects: the improvement of emergency management capacity, the appointment of cadres in the context of new challenges and missions, and the establishment collocation of prefectural-level leader teams.

## Introduction

Since the outbreak of the COVID-19 pandemic in China, government leaders have faced tremendous pressure. When facing such a large-scale test, what kind of answer sheet has been submitted? What factors affect the different achievements of different regions? This study aims to analyze the reasons for differences in performance across different regions in the context of dealing with the pandemic and to propose policy recommendations for improving governance capacity in this context. Given the central role played by the government and leading cadres in China's socioeconomic development, this study analyzes structural factors such as social, economic, and demographic factors and focuses on the ways in which the experience and characteristics of party and government-leading cadres in each region affect pandemic prevention. To compare the different requirements of pandemic prevention across party and government leaders, we compare and analyze the different roles played by the experiences and characteristics of party and government leaders of 287 prefectural-level administrative regions in the pandemic prevention model.

## Materials and methods

### Materials

**Figure 1:**
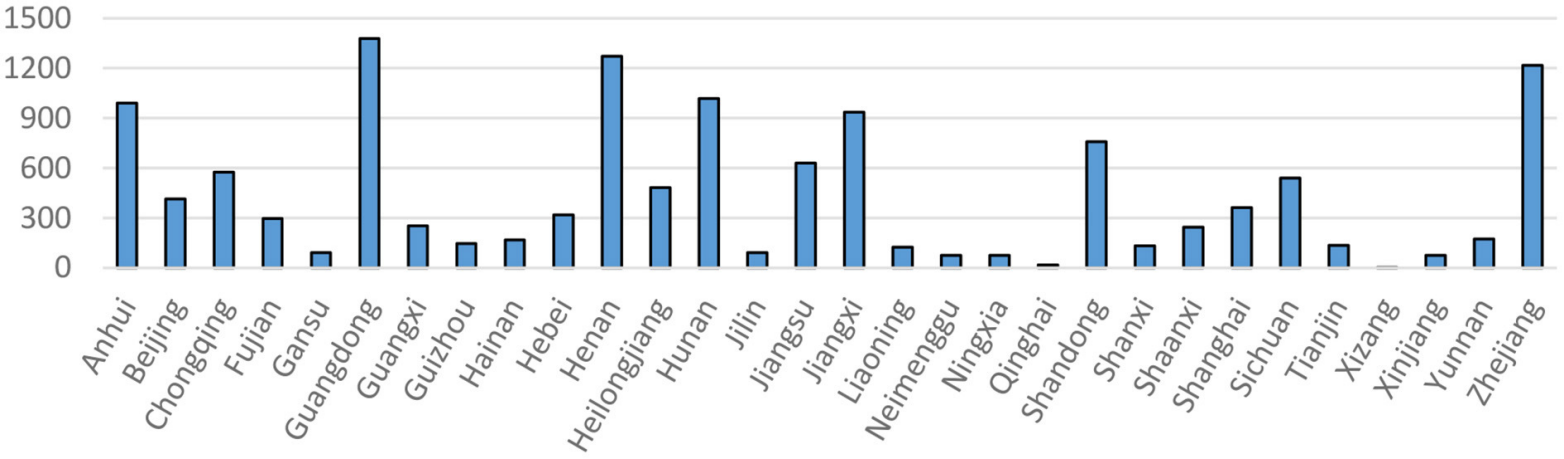
The cumulative number of confirmed cases of COVID-19 by region (as of March 18, 2020). Source: CSMAR database; China Social Quality Foundation database.

Figure 1 shows the cumulative number of confirmed cases of COVID-19 in each province apart from Hubei Province as of March 18, 2020 (when the number of new cases in mainland China reached 0 for the first time). It is evident that the cumulative number of case s was generally higher in the provinces neighboring Hubei (e.g., Henan and Hunan) and in economically developed regions (e.g., Guangdong and Zhejiang), which was likely that more people moved from Wuhan to neighboring provinces prior to January 23. In addition, economic activities and population movements in economically developed regions (including population movement from Wuhan) were more frequent and denser, thus providing conditions conducive to the spread of the pandemic.

According to a summary of 72,314 cases of COVID-19 reported by the Chinese Center for Disease Control and Prevention, Hubei Province had the highest number of confirmed cases (75%) and the highest number of cases associated with Wuhan (86%) (1). In contrast, studies on pandemic control have found that restricting population movement is a proven method that can be used to control the pandemic (2).

Based on the analysis presented above, we propose the following:

***Hypothesis 1: *** The percentage of the infected population in a region is positively associated with the population inflow from Wuhan to the region.

***Hypothesis 2: *** The percentage of the infected population in a region is positively related to the population density of the region.

***Hypothesis 3: *** The percentage of the infected population in a region is negatively related to the number of doctors in the region.

In addition to the population movement between each region and Hubei and the related socioeconomic factors, the level of health emergency management in each area is significant with respect to pandemic prevention; this factor can be measured by reference to the speed of response and the intensity of prevention measures. In Qianjiang City, Hubei Province, for example, the authorities were already preparing diligently for the pandemic prior to January 17, when 32 feverish patients were admitted and treated, and the city's leadership team decided to close the city and all entertainment venues. From February 28 until 24:00 on March 18, 2020, no new cases were reported, and work resumed in an orderly manner. Due to the swift action taken and the measures implemented, Qianjiang became one of the cities least affected by the pandemic in Hubei Province, after Shennongjia.

Based on this analysis, we propose the following:

***Hypothesis 4: *** The percentage of the infected population in a region is negatively associated with the speed of the pandemic response.

***Hypothesis 5: *** The percentage of the infected population in a region is negatively related to the intensity of pandemic prevention measures.

According to these hypotheses, leaders' abilities and qualities play a crucial role in pandemic prevention. However, leaders' implicit abilities must somehow be expressed in terms of explicit characteristics and experiences. Theoretically, upper echelon theory (UET) suggests that organizations reflect the ways in which the experiences and backgrounds of executives influence their cognition, knowledge, and skills, which in turn affect organizational decisions and thus organizational performance (3). Similarly, as the “supervisors” of a party, the personal characteristics, professional backgrounds, and experiences of local party and government leaders can reflect their abilities, thus influencing local policymaking and making a deep impact on local economic and social development (4). According to the Regulations on the Assessment of Party and Government Leading Cadres, the main content associated with the assessment of party and government cadres includes five aspects: morality, competence, diligence, performance, and integrity. In this context, “competence” includes political ability, professionalism, and organizational leadership in the process of dealing with emergencies and mass events. To a large extent, the ability of cadres is determined by their experience (5, 6). Simultaneously, officials' personal characteristics, levels of education, and experience can also influence the formulation of policies. For example, it has been shown that age, tenure and level of education affect the proportion of education spending in education policymaking (7). Furthermore, based on the public goods visibility perspective, it has been found that the tenure of both local municipal party secretaries and mayors exhibits an inverted U-shaped relationship with visible public spending (8). By analyzing the relationship between the individual characteristics of provincial environmental protection department (bureau) directors and their environmental regulatory behavior preferences, this study found that officials with experience working in environmental protection departments or those with professional study experience in environmental protection and higher levels of education tend to implement more stringent environmental regulations, which leads to the conclusion that skilled and highly educated officials are the foundation for adjusting and improving environmental regulatory behavior (9).

A comparative analysis of some typical cases indicates that the characteristics and relevant experiences (e.g., work experience in health care) of the party and government leadership teams working in each region may also impact their efforts toward pandemic prevention. For example, Shanwei city, Guangdong Province, has achieved good results regarding pandemic prevention, with five confirmed cases as of April 23, 2020 (the figure for the same period in Guangdong Province was 1,585), all of which were cured. It is easy to see that the municipal party secretary for this city was born in 1975. He has many years of grassroots work experience, having served in a significant leadership position in the Health Bureau of Lishui City, Zhejiang Province, from February 1999 to January 2003. He assumed his new post on January 11 and chaired a meeting of the Standing Committee of the Municipal Party Committee on January 21, stressing the need to strengthen leadership and press responsibility. Shanwei city achieved “zero cases” on February 28. This success indicates that experience in the health sector causes leading cadres to be more alert to pandemic prevention and provides them with relevant expertise and experience. In addition, grassroots experience across multiple regions helps leading cadres improve their implementation ability and facilitate the implementation of various prevention and control measures.

Based on this analysis, we propose the following:

***Hypothesis 6: *** The percentage of the infected population in a region is lower if the local leader has grassroots work experience.

***Hypothesis 7: *** The percentage of the infected population in a region is lower if the local leader has work experience in the central government.

***Hypothesis 8: *** The percentage of the infected population in a region is lower if the local leader has work experience in the public health sector.

***Hypothesis 9: *** The percentage of the infected population in a region is lower if the local leader has work experience in the economic department.

***Hypothesis 10: *** The percentage of the infected population in a region is lower if the local leader has work experience in a company.

***Hypothesis 11: *** The percentage of the infected population in a region is lower if the local leader has work experience in the judicial department.

***Hypothesis 12: *** The percentage of the infected population in a region is lower if the local leader team members have complementary expertise.

***Hypothesis 13: *** The percentage of the infected population in a region is lower if the local leader team has a higher level of education.

### Methods

#### Data sources

The sample referenced by this study includes 287 prefecture-level administrative regions. There are 333 prefectural-level administrative regions in China, and the following three types of regions are excluded from this study: first, prefectural-level administrative regions located in Hubei Province; second, the 26 prefectural-level administrative regions that reported zero cumulative cases, such as Sansha City and the Tibet Autonomous Region, because the cumulative number of cases in Tibet was one; and third, four municipalities that are directly under the control of the central government, because municipalities directly under the control of the central government are not comparable to other cities in terms of population density, and because pandemic prevention measures are more common in this context. This study collected information on the cumulative number of cases (excluding imported cases), economic and social indicators, the speed and intensity of the pandemic response, and the experiences and characteristics of party and government officials for each municipality as of March 18. Sources of socioeconomic data included the CSMAR database, the Wind database, the China Social Quality Foundation database, and the 2018 City Statistical Yearbook. We collected data that were unavailable from the yearbook from official sources of information such as the statistical bulletin.

To measure the speed of pandemic response, we assign a value of 3 to provinces that made specific preparations to prevent the pandemic prior to January 21 and experienced no confirmed cases prior to that time, 2 to provinces that prepared to prevent the pandemic on January 21, and 1 to provinces that took measures to prevent the pandemic after January 21. In terms of the intensity of prevention measures, provinces in which the provincial leaders conducted multiple frontline inspections within 3 days were assigned a value of 3; provinces in which the provincial leaders visited the frontline once within 3 days were assigned a value of 2, and provinces in which provincial leaders did not visit the frontline within 3 days were assigned a value of 1. In terms of response speed to the pandemic, Fujian, Guangxi, Guizhou, Henan, Ningxia, Shandong, Shanghai, Sichuan, and Zhejiang excelled, while in terms of intensity of pandemic prevention, Anhui, Beijing, Chongqing, Fujian, Guizhou, Hebei, Henan, Hunan, Jiangsu, Jiangxi, Shandong, Shaanxi, Shanghai, Sichuan, and Tianjin excelled.

Data related to the experiences and characteristics of party and government cadres were collected manually by reference to the People's Daily Online, the Party and Government Cadres Database, and official websites. Regarding the experiences and characteristics of party and government cadres, this study focuses on each administrative district; if one of the secretaries or the mayor of each city has experience in a specific area, this study considers the team to have such experience in that area. Given the large number of appointments of party and government cadres between December 2019 and January 2020, this study uses appointment and removal information reported prior to January 20, 2020, as a foundation. For cases featuring personnel changes during the pandemic, the officials who issued and implemented major policies prior to the successful control of the pandemic are counted (e.g., if an official left office during the pandemic but the pandemic was largely under control in the area prior to his departure, then information regarding that official is nevertheless used in this study). For regions in which the position of municipal party secretary or mayor was vacant, the executive deputy on the official website was used as a substitute. Information regarding officials' experiences includes various experiences prior to their assumption of their current positions.

#### Variable selection

##### Dependent variable

March 18 is when the number of new cases in mainland China reached zero for the first time. In the period afterward, there were few newly added cases in each region and using the date as a baseline to allow a more accurate comparison of the efforts made to respond to the pandemic and the corresponding performance. This study uses the cumulative number of confirmed cases per 100,000 people as of March 18 as a measure (abbreviated as cases) of the effectiveness of pandemic prevention, since the relative ratio (i.e., the number of confirmed cases per 100,000 people) is a more comparable indicator than the absolute number of cumulative confirmed cases.

##### Independent variables

The independent variables include three aspects. First, the structural socioeconomic variables include the proportion of population inflows from Wuhan, the population density, and the number of doctors per 100,000 people. The proportion of population inflow from Wuhan is measured in terms of the average proportion of people moving from Wuhan to each location between January 1 and January 23, 2020, and population density is measured in terms of the number of people per square kilometer (the correlation coefficient between population density and GDP per capita is 0.7, *p* < 0.001, so population density can, to some extent, indicate the local economic level). Second, the response of the province to the pandemic, including response speed and the intensity of measures (as described above) are taken into consideration. In this study, based on the response speed to the pandemic and the strength of measures reported for each province, values are assigned to the province's subordinate municipalities, with 3, 2, and 1 representing different speeds of response ranging from fast to slow and different strengths of measures ranging from large to small. Third, the experiences and characteristics of the party and government team, including their grassroots work experience, central government work experience, health sector work experience, economic sector work experience, enterprise work experience, and judicial sector or military work experience, are referenced. In this context, grassroots work experience refers to the experience of working in party and government organs, state-owned enterprises and institutions, villages and other economic and social organizations at the county level or below. In terms of measurement, if one of the secretaries or the mayor have experience in a certain area, this study considers the team in question to have experience in that area, which is coded as 1; otherwise, this measure is coded as 0. Regarding educational background, this study focuses on the professional heterogeneity and highest level of education exhibited by the party and government team. If the professions of two people (whoever has the highest level of education) are different, for example, if one person has an arts background and another has a science background, the team is considered to exhibit heterogeneity, which is coded as 1; otherwise, this measure is coded as 0. In this study, the highest level of education exhibited by the two leaders was defined as the highest level of education of the team. This measure includes high school and below, college, bachelor's degree, master's degree, and doctorate, which were coded from 1 to 5.

## Results

### Descriptive analysis

Table 1 shows the descriptive statistics associated with the model. The average number of cases per 100,000 people in each city in the sample was 0.96, and the average proportion of the population received as inflow from Wuhan was 0.086%, with more than half of the Wuhan outflow population traveling to other cities in Hubei Province.

**Table 1:** Descriptive statistics of variables related to pandemic prevention.

	***M***	**SD**	**Cases**	**Inflow**	**DOP**	**NOD**	**Speed**	**Intensity**
Cases	0.96	1.391	1					
Inflow	0.086	0.166	0.366^**^	1				
DOP	436.213	534.753	0.435^**^	0.385^**^	1			
NOD	242.932	109.666	0.403^**^	0.268^**^	0.513^**^	1		
Speed	2.049	0.805	−0.04	0.150^*^	0.05	−0.178^**^	1	
Intensity	2.638	0.556	−0.123^*^	0.122^*^	−0.02	−0.143^*^	0.477^**^	1
Grassroots	0.798	0.402	−0.128^*^	0.082	−0.004	−0.029	0.085	0.172^**^
Central	0.178	0.383	0.160^**^	0.029	0.051	0.029	0.051	−0.074
Health	0.077	0.267	−0.130^*^	−0.108	−0.052	0.03	−0.066	−0.001
Economic	0.537	0.5	−0.005	−0.017	−0.002	0.014	0.083	0.124^*^
Company	0.418	0.494	−0.055	0.031	0.064	0.112	−0.043	0.032
Judicial	0.338	0.474	−0.043	−0.078	−0.018	−0.03	−0.053	−0.011
Expertise	0.359	0.481	−0.071	0.002	0.051	0.016	−0.036	−0.009
Education	4.38	0.528	−0.035	0.06	0.151^*^	0.059	0.014	−0.042
	**Grassroots**	**Central**	**Health**	**Economic**	**Company**	**Judicial**	**Expertise**	**Education**
Grassroots	1							
Central	−0.084	1						
Health	0.112	−0.065	1					
Economic	−0.067	−0.025	0.005	1				
Company	−0.066	−0.024	−0.005	0.009	1			
Judicial	0.011	0.034	−0.04	0.014	0.051	1		
Expertise	−0.094	0.032	0.03	−0.062	0.043	−0.074	1	
Education	−0.197^**^	0.115	−0.059	0.086	0.019	−0.054	0.109	1

** p < 0.01;

* < 0.05. Cases, Inflow, DOP, NOD, Speed and Intensity refer to the cumulative number of confirmed cases per 100,000 persons as of March 18, the population inflows from Wuhan, the density of the population, the number of doctors per 100,000 people, the response speed, and the response intensity, respectively. Grassroots, Central, Health, Economic, Company and Judicial refer to work experience in the corresponding department. Expertise refers to the different expertise of different leader team members. Education refers to the highest level of education within the leader team.

In terms of pandemic response, most provinces responded to the pandemic with medium or higher levels of speed and intensity. Among the various types of officials' experience, the mean value of grassroots experience is 0.8, indicating that most party and government teams have grassroots experience; party and government teams with central government work experience account for 17.8% of the total sample, and further analysis of these municipalities in this study reveals that they are distributed in the central-eastern region, which is located closer to Hubei Province. In terms of functional backgrounds, party and government teams with experience in the health sector account for 7.7% of the sample; in contrast, those with experience in the economic sector account for more than half of the sample, which is in line with China's focus on economic development in recent decades; the proportion of party and government teams with corporate and judicial experience is approximately 40%. Regarding educational background, leader teams featuring different (or complementary) areas of study account for more than one-third of the sample, while the average highest level of education of the party and government teams is possession of a master's degree or above. The results regarding the correlations among the variables indicate that the proportion of the population moving into the city from Wuhan, the population density, the proportion of doctors, and the intensity of the measures taken by the provinces in question in response to the pandemic are significantly negatively correlated with the number of cases, a finding which is in line with the expectations of this study. Regarding the experiences and characteristics of the party and government team, grassroots experience, health experience, and corporate experience were all negatively correlated with the number of cases, while central government work experience was positively correlated with the number of cases; professional heterogeneity and highest education were all significantly negatively correlated with the number of cases.

### Regression analysis

A hierarchical regression model was conducted to examine the explanatory power of socioeconomic factors, pandemic response speed and intensity factors as well as the experiences and characteristics of the party and government teams in each city with respect to the number of confirmed cases per 100,000 persons. The results are shown in Table 2. Hypotheses 1, 2, 3, 6, 8, 10, 12, and 13 were supported, while the other hypotheses were not supported.

**Table 2:** Regression analysis.

**Number of confirmed cases per 100,000 persons**							
	**Variable**	**Model 1**	**Sig**.	**Model 2**	**Sig**.	**Model 3**	**Sig**.
Social-economic factors	Inflow	1.800	0.000	1.960	0.000	1.949	0.000
	DOP	0.001	0.000	0.001	0.000	0.001	0.000
	NOD	0.003	0.000	0.003	0.001	0.003	0.001
Response capabilities	Speed			0.012	0.909	−0.039	0.691
	Intensity			−0.302	0.038	−0.188	0.187
Experiences and characteristics of the leader team	Grassroots					−0.509	0.004
	Central					0.484	0.007
	Health					−0.433	0.094
	Economic					0.027	0.846
	Company					−0.280	0.044
	Judicial					−0.086	0.549
	Expertise					−0.258	0.071
	Education					−0.379	0.005
Statistical parameters	*R* ^2^	0.272		0.285		0.36	
	Adjusted *R*^2^	0.264		0.273		0.33	
	*R*^2^ Change	0.272		0.013		0.075	
	*F* Change	35.264 (0.000)		2.59 (0.077)		4.002 (0.000)	
	*N*, df	287, 3		287, 5		287, 13	

Significance levels are listed in parentheses.

Specifically, according to Model 1, the higher the proportion of the Wuhan population that moved into a city from January 1 to January 23, the higher the number of cases in that city, with the number of cases increasing by 1.8 as a result of a one percent increase in the proportion of people moving into the city from Wuhan. Since the average number of cases in the sample was only 0.96, the intensity of the effect of the population moving from Wuhan was very significant.

Combined with the fact that the first confirmed cases in each province had a history of exposure to Wuhan, this result once again illustrates the necessity and correctness of the Wuhan closure policy. The number of cases is also higher in cities with a high population density. The regression analysis indicates that an increase of 1 person per square kilometer of population density increases the number of cases by 0.001, which reflects the characteristics of the pandemic and indirectly indicates the significance of maintaining social distancing with respect to pandemic prevention. The more doctors per 100,000 people, the higher the number of cases diagnosed in the area. An increase of 1 doctor per 100,000 people leads to an increase of 0.003 cases because the number of doctors is an indicator of local economic and social development and because a significant portion of the outflow population from Wuhan travels to economically developed areas. In terms of the response speed and intensity, as shown in Model 2, the higher the intensity of the measures, the lower the number of cases, and an increase in the intensity level results in 0.302 fewer cases in the city in question; however, the response speed does not have a significant effect on the number of cases, likely due to the fact that most of the areas that exhibited a fast response were provinces neighboring Hubei (e.g., Henan Province) and economically developed areas (e.g., Guangdong Province). The rapid spread of the pandemic decreased the effect of fast response speed to a greater extent; in contrast, regions with slow response speed, which are often located farther from Hubei (e.g., Inner Mongolia Autonomous Region), faced relatively easy prevention situations.

After including the experiences and characteristics of party and government teams in Model 3, the explanatory power of the dependent variable increased by 7.5%. Specifically, the grassroots experience of party and government officials in each city was significantly negatively associated with the number of cases, such that the number of cases in a city in which the leadership team had grassroots experience was 0.509 lower than in a city in which the leadership team did not have grassroots experience. Possible reasons for this finding are that officials with grassroots experience are more familiar with the country's situation and can develop policies that are suitable for local realities. Work experience in the central government was significantly positively correlated with the number of cases, with the proportion of cases in a city in which the party and government team had such experience being 0.484 higher than in other cities, probably since most such cities are located closer to Hubei and were thus more influenced by the influx of people from Wuhan.

Health sector experience was significantly negatively associated with the number of cases, with 0.433 fewer cases being reported in a city in which the party and government team had health sector experience than in other cities, a finding which was in line with previous conjectures made by this study as well as with the findings of recent studies on executive teams (10, 11). Through a literature review, researchers have found that over the past decade, the professional background and overseas experience of executive teams have begun to become more relevant, as these characteristics can more accurately describe the cognitive characteristics and skill composition of teams, which can in turn better predict organizational performance (12). In the pandemic prevention process, officials with experience in the health sector tend to have higher sensitivity as well as more specialized knowledge with respect to the application of precise measures.

The number of cases was significantly negatively correlated with corporate experience, with 0.28 fewer cases being reported in a city in which party and government teams had corporate work experience than in other cities, probably since officials with corporate work experience are more sensitive to various types of social and market information. Specifically, compared to government management, corporate management involves a rapidly changing, competitive and uncertain environment, which requires leaders to make timely changes. Officials with corporate experience may also be more pragmatic and may thus react more quickly and effectively in the context of pandemic prevention. In terms of educational background, professional heterogeneity was significantly negatively associated with the number of cases, with the number of cases in a city in which the party and government team had different professions being 0.258 less than in other cities, a finding which is consistent with the main claims of information decision theory (13). This theory suggests that the heterogeneity of team members facilitates the integration of different types of information and mutual learning among members, leading to the development of novel perspectives and thus enhancing the quality of team decisions and improving team performance (14). The highest level of education of the team leaders is significantly negatively related to the number of cases, and for each increase in the highest level of education among the team, such as from a bachelor's to a master's degree, the number of cases decreases by 0.379, a conclusion which is consistent with previous findings (15, 16). The higher the level of education exhibited by the leader, the better the economic performance becomes, since higher educational attainment can represent knowledge and skills to some degree (17). In the context of the pandemic, the ability to study and judge the pandemic and formulate prevention policies places higher demands on the governance capacity of officials, and officials with higher levels of education are more likely to have a comprehensive knowledge base and theoretical system, which has positive implications regarding the prevention of the pandemic.

**Figure 2:**
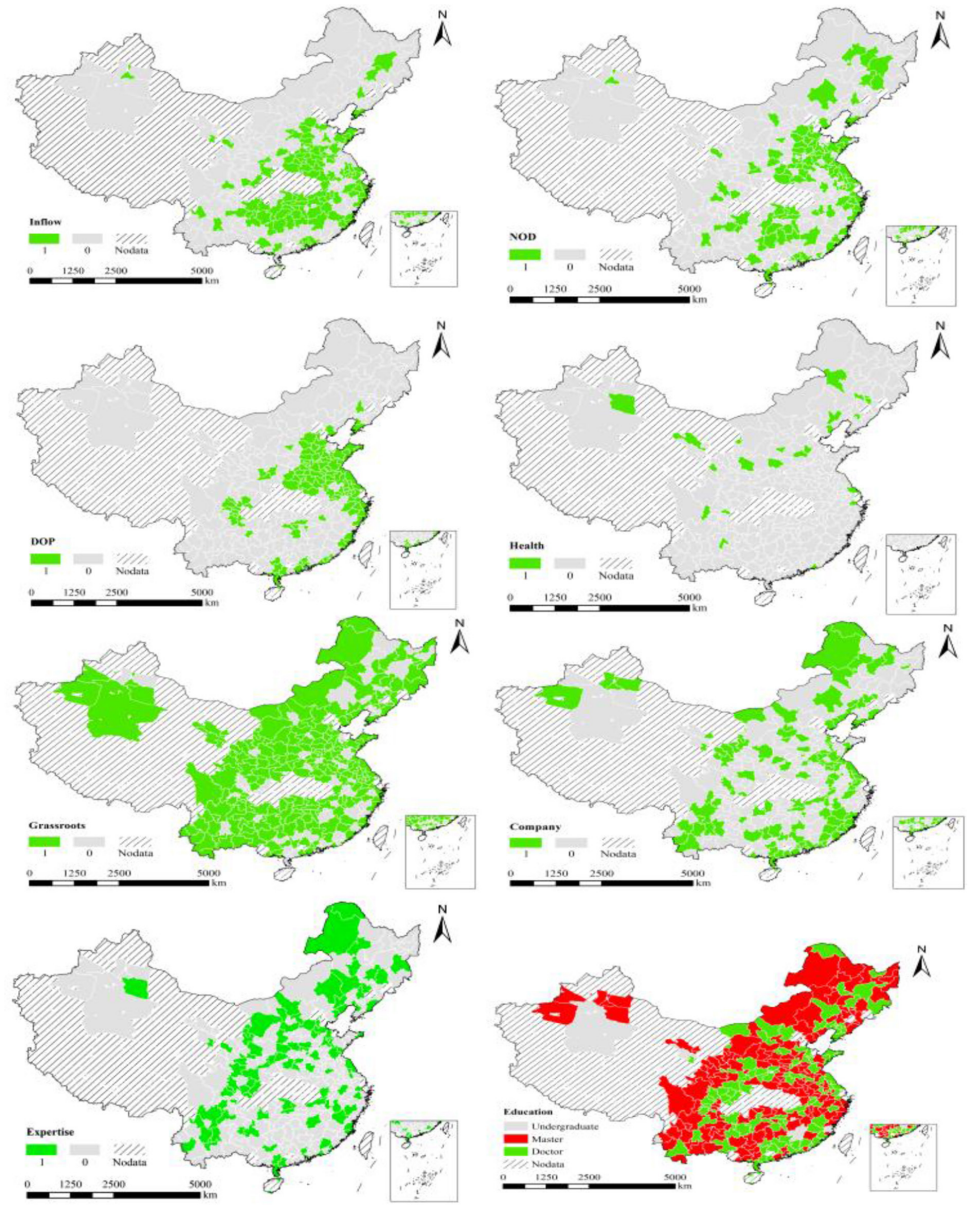
Results in visual map. Inflow, DOP, NOD refer to the population inflows from Wuhan, the density of the population, the number of doctors per 100,000 people respectively. Grassroots, Health, Company refer to work experience in the corresponding department. Expertise refers to the different expertise of different leader team members. Education refers to the highest level of education within the leader team.

To visualize the results, we have drawn maps by ArcGIS software in Figure 2. The eight maps show eight factors that significantly affect pandemic prevention. Specifically, in the “inflow” map, the cities with above-average inflow population from Wuhan are indicated in green, which clearly shows that the cities around Wuhan and the economically developed cities have more inflow population and face a more serious pandemic. In the “DOP” and “NOD” maps, the cities with above-average population density and doctors are marked in green, and their cases are higher than the others. In the “Health”, “Grassroots”, and “Company” maps, the cities where the local leader team own such kind of experience are indicated in green, which verifies these cities are more successful in dealing with the pandemic. And leader teams with health sector work experience are scarce in contrast with the grassroots work experience, while the cities where local leader teams own company work experience show a widespread distribution. Further, in the “Expertise” map, the cities where local leader teams with complementary expertise are marked in green and are dispersed. The last map illustrates the education levels of political leader team are high, and the cities where leader teams own doctor degree, marked in green, are more successful in the pandemic prevention.

## Discussion

Based on the analysis of the factors influencing the performance of different regions in this “large-scale test” according to the socioeconomic level of each region, the characteristics and experiences of the party and government team have a tremendous impact on the prevention of the pandemic. According to the empirical results, with the aim of proposing directions for future development, this study proposes policy recommendations regarding the appointment of party and government cadres, the establishment of party and government teams, and the improvement of government emergency governance capacity.

First, regarding the training and appointment of party and government cadres, highly educated personnel should be focused on grassroots-level departments. This study finds that party and government teams with grassroots experience and higher levels of education have a positive effect on pandemic prevention. Therefore, the selection of cadres should take grassroots experience into account and encourage cadres with higher education or even overseas experience to “sink” with the aim of sharpening their wills and improving their skills while familiarizing themselves with the national situation. The theoretical foundation and knowledge reserves should be consolidated, and knowledge should be applied to and improved in practice.

Second, with respect to future changes in government tasks and the focus of social governance, workers with different functional backgrounds and relevant experiences should be selected to join the leadership team. The pandemic is still highly spread in the whole global (18), while fewer cases are observed in cities of China in which party and government teams have work experience in the health sector and in which the proportion of party and government cadres with such experience does not exceed 10%. If the talk of dealing with public health problems becomes a regular responsibility for social governance in the future, the proportion of cadres with backgrounds in the field of health should be appropriately expanded in the selection of cadres. An emergency command consisting of and led by key leaders from the Health and Welfare Commission with the assistance of relevant experts should be established. In addition, corporate experience has positive significance with respect to pandemic prevention; therefore, the exchange of cadres should be encouraged to enhance the diversity of their experience and their comprehensive ability.

Third, regarding the matching of the party and government leadership team, cadres with different professional backgrounds and experiences should be selected to form a team featuring complementary advantages. Different professional backgrounds imply different knowledge and skill backgrounds and different ways of thinking, which can facilitate the integration of a variety of information and thus improve the quality of team decision-making; similarly, different experiences result in different experiences and abilities, and diverse knowledge and experiences match the governments multitask-focused and highly complex goal system, which is consistent with certain previous findings (19, 20). For example, at least one leader in the party and government team should have grassroots work experience to ensure that that the team can tailor its work to local conditions when formulating and implementing various policies; at least one leader in the party and government team should have a higher level of education to provide a more open-eyed and open-minded perspective, which is conducive to improving the quality of decision-making. When responding to unexpected events, different responsibilities should be assigned to party and government cadres based on their abilities and backgrounds. In the face of the main development objectives, i.e., economy development and social stability (21), the appointment of cadres and the division of work should be adjusted in a flexible manner. In other words, the matching of party and government teams should consider the professional quality of cadres but also examine the learning ability of cadres, focus on the comprehensive experience of cadres, and promptly make adjustments to promote competent cadres based on the requirements of changing tasks.

## Conclusions

Based on the analysis discussed above, the following conclusions can be drawn from this study.

First, both the structural factors and the subjective initiatives associated with each location affect pandemic prevention. For example, the factors influencing the prevention of the pandemic include the proportion of the population from Wuhan, the population density, and the proportion of doctors, all of which are structural factors reflecting local socioeconomic conditions; simultaneously, the progress of pandemic prevention is influenced by response speed, the intensity of measures, and the level of governance capacity as represented by the experiences and characteristics of the party and government teams.

Second, the experiences, functional backgrounds, and levels of education of members of the party and government team have significant effects on pandemic prevention. The regression analysis regarding pandemic prevention found that, based on the structural factors (Model 1) and response measures (Model 2) for each region, the introduction of the experiences and characteristics of the party and government team (Model 3) greatly improved the explanatory power of the model, accounting for approximately 26% (0.075/0.285) of the variance in the dependent variable (variance); in addition, the F variation was significant.

Third, grassroots experience and the highest level of education among party and government leaders have significant positive impacts on pandemic prevention. Grassroots experience helps cultivate leaders' sensitivity to national conditions and the environment and helps test their practical operation ability and execution ability. Simultaneously, the highest level of education among the local and municipal party and government teams has positive significance for pandemic prevention, indicating that the knowledge and perspectives of leading cadres have positive effects on broadening their thinking and improving their ability to solve complex problems, once again verifying the importance of the intellectualization of leading cadres.

Due to the limitations of data availability, the analysis conducted by this study faces certain limitations, such as the facts that the characteristics of leaders can be measured only in terms of their explicit experiences and that information that can truly reflect the responsibility and competence of leaders is difficult to obtain in a comprehensive manner. Nevertheless, the conclusions drawn from this study following rigorous analysis and careful consideration have both theoretical and practical significance.

## Data availability statement

The data in this article is from publicly available data sources: CSMAR, Wind, China Social Quality Foundation, 2018 City Statistical Yearbook and People's Daily Online.

## Ethics statement

The studies involving human participants were reviewed and approved by School of Management, Henan University of Technology. The patients/participants provided their written informed consent to participate in this study.

## Author contributions

JL: methodology, software, formal analysis, investigation, writing—original draft preparation, and supervision. JZ and ZH: conceptualization, validation, resources and data curation, review, and editing. All authors contributed to the article and approved the submitted version.

## Funding

This work was funded by the National Natural Science Foundation of China (Grant Number 7217020419), National Social Science Foundation of China (Grant Number 21BGL140), Research Project of Humanities and Social Sciences for Universities in Henan Province (Grant Number 2023-ZDJH-120), and Henan University of Technology High-level Talent Research Start-up Fund Project (Grant Number 2021SBS34).

## Conflict of interest

The authors declare that the research was conducted in the absence of any commercial or financial relationships that could be construed as a potential conflict of interest.

## Publisher's note

All claims expressed in this article are solely those of the authors and do not necessarily represent those of their affiliated organizations, or those of the publisher, the editors and the reviewers. Any product that may be evaluated in this article, or claim that may be made by its manufacturer, is not guaranteed or endorsed by the publisher.

## References

[1] Wu Z Mcgoogan JM. Characteristics of and important lessons from the coronavirus disease 2019 (COVID-19) outbreak in China: Summary of a report of 72 314 cases from the Chinese center for disease control and prevention. JAMA. (2020) 323:1239–42. 10.1001/jama.2020.2648 32091533

[2] Wu JT Leung K Leung GM. Nowcasting and forecasting the potential domestic and international spread of the 2019-nCoV outbreak originating in Wuhan, China: a modelling study. Lancet. (2020) 395:689–97. 10.1016/S0140-6736(20)30260-9 32014114 PMC7159271

[3] Hambrick DC Mason PA. Upper echelons: the organization as a reflection of its top managers. Acad Manage Rev. (1984) 9:193–206. 10.5465/amr.1984.4277628

[4] Fanghua Q Shan W. An empirical analysis of the relationship between personal characteristics of municipal party secretaries and GDP growth–a data analysis based on 1000 samples of municipal party secretaries in 10 years in the top 100 GDP cities. J B Inst Adm. (2019) 4:1–8. 10.16365/j.cnki.11-4054/d.2019.04.001

[5] Lin TJ. An analysis of the economic logic of mayoral promotion in prefecture-level cities in China. Public Manage Res. (2007) 0:45–68.

[6] Luo DL She GM Chen J. The correlation between economic growth performance and promotion of local officials revisited: new theory and new evidence based on prefecture-level city data. Econ Q. (2015) 3:1145–72. 10.13821/j.cnki.ceq.2015.03.014

[7] Song R Chen GH. Official characteristics, experience and local government education expenditure preferences-empirical evidence from Chinese prefecture-level cities. Econ Manage. (2016) 12:149–69. 10.19616/j.cnki.bmj.2016.12.011

[8] Wu M Zhou LA. Promotion incentives and city construction: A public goods visibility perspective. Econ Res. (2018) 12:97–111.

[9] Han C Liu XY Wang H. Incentives and behavioral preferences of regulatory officials-a new solution to environmental regulation failure under the lack of independence. Manage World. (2016) 2:82–94. 10.19744/j.cnki.11-1235/f.2016.02.010

[10] Cannella Jr AA Park J Lee H. Top management team functional background diversity and firm performance: examining the roles of team member colocation and environmental uncertainty. Acad Manage J. (2008) 51:768–84. 10.5465/AMJ.2008.33665310

[11] Carpenter MA Geletkanycz LMA Sanders LWG. Upper echelons research revisited: antecedents, elements, and consequences of top management team composition. J Manage. (2004) 30:749–78. 10.1016/j.jm.2004.06.001

[12] Zhang SB Li Y. Top management team research: progress, comparison and direction. Q J Manage. (2018) 2:85–112.

[13] Simons T Pelled LH Smith KA. Making use of difference: diversity, debate, and decision comprehensiveness in top management teams. Acad Manage J. (1999) 42:662–73. 10.5465/256987

[14] Lee H Park J. The influence of top management team international exposure on international alliance formation. J Manage Stud. (2008) 45:961–81. 10.1111/j.1467-6486.2008.00772.x

[15] Dreher A Lamla MJ Lein SM Somogyi F. The impact of political leaders' profession and education on reforms. J Comp Econ. (2009) 37:169–93. 10.1016/j.jce.2008.08.005

[16] Besley T Montalvo JG Reynal-Querol M. Do educated leaders matter? Econ J. (2011) 121:205–27. 10.1111/j.1468-0297.2011.02448.x

[17] Spilerman S Lunde T. Features of educational attainment and job promotion prospects. Am J Soc. (1991) 97:689–720. 10.1086/229817

[18] Ding D Zhang R. China's COVID-19 control strategy and its impact on the global pandemic. Front Public Health. (2022) 10:857003. 10.3389/fpubh.2022.857003 35359767 PMC8964044

[19] Zhu WX Zhang P Li PF Zi ZY. Predicament of micro, small and medium enterprises and policy efficiency improvement under pandemic shock-analysis based on two national questionnaire surveys. Manage World. (2020) 4:13–25. 10.19744/j.cnki.11-1235/f.2020.0049

[20] Xiao MZ Shi HY. Research on the cultivation and development mechanism of party and government leading cadres in the new era - taking leading cadres at the provincial and ministerial levels as an example. J Chin Acad Gov. (2018) 5:100–7. 10.14119/j.cnki.zgxb.2018.05.013

[21] Zhang R Worden S Xu J Owen JR Shi G. Social stability risk assessment and economic competitiveness in China. Hum Soc Sci Commun. (2022) 9:309. 10.1057/s41599-022-01329-835915306

